# Independent mobility, perceptions of the built environment and children's participation in play, active travel and structured exercise and sport: the PEACH Project

**DOI:** 10.1186/1479-5868-7-17

**Published:** 2010-02-19

**Authors:** Angie S Page, Ashley R Cooper, Pippa Griew, Russell Jago

**Affiliations:** 1 Department of Exercise, Nutrition & Health Sciences, University of Bristol, Bristol, UK

## Abstract

**Background:**

Independent mobility (IM) and perceptions of the built environment may relate differentially to children's participation in various physical activity contexts. This cross-sectional study investigated whether independent mobility and perceptions of the built environment in boys and girls were related to physical activity in three different contexts (outdoor play, structured exercise/sport, active commuting).

**Methods:**

Thirteen hundred and seven 10-11 year old boys and girls from 23 schools in a large UK city completed a computerised questionnaire. Independent variables in logistic regression analyses were weekly self-reported frequency of participation in outdoor play, structured exercise/sport and mode of travel home from school. Dependent variables were perceptions of the environment (aesthetics, nuisance, safety, social norm, constraint, play space, accessibility), local and area independent mobility and linear distance from home to school. Analyses were adjusted for body mass index, minutes of daylight after school, level of neighbourhood deprivation and pubertal status.

**Results:**

For boys, local independent mobility (Local-IM) was related to an increased likelihood of everyday participation in play (OR 1.58: 95% CI 1.19-2.10), structured exercise/sport (OR 1.42: 1.06-1.89) and active commuting (OR 1.40: 1.07-1.87) but was only related to active commuting for girls (OR1.49: 1.07-2.07). Boys and girls were more likely to report playing out every day if they had higher scores for Social Norm (Boys: OR 1.63 (1.12-2.37); Girls: OR 1.53 (1.01-2.31)) and, for girls only, more positive perceptions of traffic safety (OR 1.63: 1.14-2.34). Easy access to a range of destinations was the dominant predictor for taking part in structured exercise/sport everyday (Boys: OR 1.62 (1.01-2.66); Girls: OR 1.65 (1.07-2.53)). Shorter distance from home to school (OR 0.99: 0.98-0.99) and, for boys only, greater perceived accessibility (OR 1.87: 1.04-3.36) were significantly related to active commuting to school.

**Conclusions:**

Perceptions of the physical environment relate differently to different physical activity contexts and by gender. The only consistent correlate for outdoor play, structured ex/sport and active commuting was higher independent mobility to visit local destinations (Local-IM) for boys. Considering both the physical activity context and its independent correlates should improve the specificity of physical activity interventions in children.

## Introduction

Regular physical activity in children is associated with many physical and mental health benefits [1] and inactivity has been strongly implicated in the rise of childhood obesity [2]. Effective interventions to increase physical activity in children beyond the school setting are limited [3,4]. One reason for this may be that physical activity behaviour and its associated determinants may be different in contrasting physical activity contexts or context specific behaviours [5]. Physical activity context is not clearly defined in the literature but the key components are likely to include type of activity, location or setting, time period, and who takes part [6,7]. For example, children's participation in Physical Education at school, where volition and choice are limited, may differ from participation in free play both within and beyond the school gates.

Giles-Corti and King (2009) suggest that most individuals obtain physical activity from more than one context, and this is desirable as different types of activity provide different health and social benefits [1]. Increased active commuting in children is associated with increased cardiovascular fitness [8] whereas structured sport and team based activities are associated with increased physical competence and social benefits [9]. Child instigated 'free' play is also associated with unique social and developmental benefits [10,11].

There is an absence of temporal data but it seems likely that physical activity in certain contexts may have declined whereas others have been maintained or even increased. For example, the UK National Travel Survey indicates that the proportion of trips undertaken on foot by children declined from 47% to 32%, between 1985/6 and 2002 [12] and Sturm [13] reported that time in unstructured activities including play decreased from 1981 to 1997 in the U.S. whilst time spent in structured sport and school/childcare increased in the same period. This has led some authors to suggest that children's ability to take part in unstructured outdoor activity has fallen as a consequence of children's increasingly 'structured, supervised and indoor lives' [14]. This is of concern as lower levels of time spent outdoors is related to lower physical activity [15] and higher levels of obesity [16].

Determinants of activity are also likely to vary in different activity contexts [5]. Factors associated with outdoor free play may relate more to play space, friendship groups, local traffic and ability to play unsupervised [17,18] whereas correlates of structured sport and exercise may be more related to car ownership, household income, and access to specific sports and exercise facilities [15,19]. For active commuting to school the distance from home to school is an important correlate alongside parental perceptions of the convenience of active commuting [20]. Environmental correlates, both objective and perceived, could be particularly important in different contexts as they may be independent of, or partially mediate, other correlates to fully explain children's physical activity behaviour [21]. Participation in different physical activity contexts as well as their determinants may also vary by socio-economic position. It has been reported that those from lower socio-economic position have less access to structured sport/exercise but more access to free play [19,22].

Understanding the different correlates of behaviour specific contexts may be particularly important in the transition from primary to secondary school because opportunities for physical activity may change as different facilities become available or distance from home to school increases. This age of transition (10-12 years) is characterised by a downward shift in physical activity and an increase in sedentary behaviour [23]. This period is also a time when independent mobility (children's ability to move around in their neighbourhood unaccompanied by an adult) changes [24]. We have shown that children with greater independent mobility engage in a greater volume of physical activity around this transition period [25]. Limited data are available but it is likely that independent mobility relates to perceptions of the environment. For example independent mobility might be higher where there is low traffic density, greater personal safety, less crime or where it is more common to see other children playing unsupervised.

Objective measures such as accelerometers have been crucial in establishing a stronger case for the link between volume of physical activity and health outcomes in young people [26,27] but they are limited in their ability to describe the mode or context of physical activity [28,29]. Temporal patterning of accelerometer data has been used to identify key time periods where group differences in physical activity are seen in relation to obesity status [30] and active commuting [31]. However these data are limited because they provide no information about what the children are doing in these time-periods and thus what the specific behavioural target for intervention should be. This study sought to address these issues by investigating whether independent mobility and perceptions of the environment were related to frequency of participation in three different physical activity contexts: outdoor play, structured sport/exercise and mode of travel to school.

## Methods

This study used baseline data from the PEACH project (Personal and Environmental Associations with Children's Health). The PEACH project is a longitudinal study designed to investigate the environmental and personal determinants of physical activity, eating behaviours and obesity in young people as they transition from the final year of primary school (aged 10 to 11 years) to the first year of secondary school (11 to 12 years).

### Participants

Thirteen hundred and seven year 6 children (aged 10-11 years) were recruited from 23 of 72 state funded primary schools within a large UK city between September 2006 and July 2008. The primary schools were selected as they had the highest transition rates (>40%) to eight local state funded secondary schools. These eight urban secondary schools were selected on the basis of neighbourhood deprivation and geographic location to represent the city. Neighbourhood deprivation was assessed using Index of Multiple Deprivation (IMD), a composite score based on seven categories of deprivation (income, employment, health and disability, education skills and training, housing, and geographical access to services) [32]. Index of Multiple Deprivation scores were derived from the school post-code and a higher score (and lower rank) indicates a higher level of deprivation. Compared to IMD values for England, the primary schools in this study were located in relatively deprived areas (8 of the 23 schools were ranked in the lowest 20% for IMD). Only one primary school approached declined to take part in the study.

### Procedure

Schools were contacted by phone and/or by letter to invite them to take part in the study. For those schools who agreed to participate, Year 6 school children and teachers were briefed about the study and provided with an information pack to take home. On measurement days, only children who provided written parental consent were invited in small groups (4-6 children) to take part. Children had their height and weight measured in a private room by the researcher and then completed a computerised self-report questionnaire with a researcher nearby if help was required. Children were given an accelerometer to take home and instructed to wear it for seven days and then bring it back to school. This study was approved by a University Ethics Committee.

### Measures

#### Dependent variables

Frequency of participation in three different physical activity contexts was measured by child self-report on a computerised questionnaire. Descriptors and pictures were used to describe each context prior to the children completing the question for each physical activity context. The three physical activity contexts were:

##### Outdoor play

Children were asked '*How often do you normally play out?' *Playing out was described as things like riding a bike, kicking a ball around, skipping, jumping/running around, skateboarding, riding a scooter and activities that make you move around but are not structured. Children could select from one of seven responses ranging from every day to hardly ever.

##### Exercise/sport (Ex/sport)

Children were asked 'How *often do you normally exercise or play sport*? This was described as organised sports, team sports or exercise activities like martial arts, swimming, playing tennis, football or other team games, dance lessons, ice skating, horse riding. Children could select from one of seven responses ranging from every day to hardly ever.

##### Mode of travel home from school

Children were asked '*How do you usually get home from school*'. Options were walk, cycle, car, bus or train.

The three self-report items were reverse coded so that a higher score represented higher frequency of activity behaviour. Self-report items were positively correlated with objectively measured physical activity (GT1M Actigraph, FL, USA: average weekly accelerometer counts per minute; play (r = 0.138, p = 0.001), exercise/sport (r = 0.153, p = 0.001), mode of travel (r = 0.167, p = .0.001). Methods and data reduction for accelerometry data have been described elsewhere [25]. Intra class correlations [33] on a sample of children from the same city (n = 46) over a two week period were generally good ranging from 0.800 (p = 0.001) for play, 0.703 (p = 0.001) for active travel and 0.407 (p = 0.001) for structured ex/sport.

#### Independent variables

All independent variables were measured using a self-reported computerised questionnaire. Table 1 provides a summary of measures representing children's self-reported independent mobility and perceptions of their environment.

**Table 1:** Summary of independent mobility and environmental measures

Subscale name	No. items	Content	Response format 4 choices	Mean Gender diff**	SD	Alpha	ICC	Source Ref
Local-IM	4	Go unsupervised to *local shops, school, friends, park*	Never to Always	3.04**	0.76	0.70	0.81	25
Area-IM	7	Go unsupervised to *shopping & sports centre, pool, library, cinema, bus stop, arcade*	Never to Always	1.91**	0.75	0.79	0.78	25
Aesthetics	4	Perceptions of problem of *litter, graffiti, vandalism, dog fouling*	Strongly Disagree to Strongly Agree*	2.29	0.78	0.81	0.69	
Nuisance	3	Perceptions of *crime, noise, bullying *in local neighbourhood	Strongly Disagree to Strongly Agree*	2.78	0.64	0.61	0.65	
Personal safety	4	Perceptions of *safety at night, daytime, fear of strangers, dark*	Strongly Disagree to Strongly Agree	3.00**	0.57	0.67	0.42	34
Traffic safety	4	Perceptions of *safe places to cross, heavy traffic, roads, pollution*	Strongly Disagree to Strongly Agree*	2.48**	0.60	0.56	0.48	34
Access(1)	6	Ease of access to *shops, park, bus stop, library, sports & shopping centre*	Very easy to very difficult	3.10	0.42	0.56	0.59	35
Access(2)	2	Easy of access to *school, best friend's house*	Very easy to very difficult	3.35	0.65	0.56	0.69	35
Social Norm	4	*Children to play with, children on streets, people walking and cycling around*	Strongly Disagree to Strongly Agree	2.93	0.54	0.60	0.78	34
Playspace	2	*Space to play inside/outside*	Strongly Disagree to Strongly Agree	2.97	0.66	0.51	0.81	ND
Constraint	2	Want to get outside to play/stuck inside	Strongly Disagree to Strongly Agree*	2.18**	0.77	0.63	0.77	ND

**Note**: * Items recoded so that higher score indicates more positive perception of the environment, ** boys scores significantly higher than girls (independent t-test).

**Abbreviations**: ICC (intra-class correlation), SD (Standard deviation), ND (newly derived)

##### Independent mobility (IM)

Independent mobility was assessed using the stem '*How often are you allowed to go to the following places on your own or with friends (without an adult)'*. From these 11 items, two subscales were derived - Local independent mobility (**Local-IM **which included best friend's house, school, local shops and park or playground) and Area Independent Mobility (**Area-IM**: swimming pool, library, cinema, arcade, bus stop, sports and shopping centre). Full details of subscale development have been previously reported [25].

#### Perceptions of the physical environment

##### Aesthetics, Nuisance, Safety, Social Norm, Constraint

Twenty three items were included in the computerised questionnaire to measure children's perceptions of their environment in '*the area where I live (my neighbourhood*)'. Nineteen of these items were based on existing measures [34] developed to measure aesthetics, nuisance, personal and traffic safety and social norm for physical activity (see Table 1 for content). In addition four items were included which were hypothesised to measure environmental constraint, i.e. the child's perception that they were restricted from being active by their physical environment. These items were *'I often feel I want to get out of the house to get some space to play', 'I think there is a lot of space for me to play outside', 'I often feel stuck inside when I would rather go out to play' *and *'I think there is a lot of space for me to play inside'*. For each of the 23 items, children could select from one of four options (Strongly Disagree to Strongly Agree).

As the items hypothesised to represent perceptions of the environment were significantly correlated, principal components analysis (PCA) with Varimax rotation was conducted to reduce the items into associated components. The resulting scree plot and Eigen values were interpreted and factors selected. This process resulted in seven factors which were a). **Aesthetics **explaining 19.92% of the variance; b). **Personal safety **explaining 11.21% of the variance, c). **Nuisance **explaining 7.37% of the variance; d). **Social Norm **explaining 6.20% of the variance and e). **Traffic safety **explaining 5.87% of the variance. All items loaded highest on their intended factor with no cross-loadings over 0.4 The four new items separated into two separate factors - **Playspace **explaining 4.47% of the variance (two items -space to play inside and outside) and **Constraint **explaining 5.10% of the variance comprising the other two items (desire to get out and play and feeling stuck inside). PCA were similar for boys and girls so generic means were generated for each factor and used in subsequent analyses. Intra-class correlation for these scales with a sub-sample of children (n = 46) from the same city over a two week period were generally moderate to good (see Table 1 for subscale summary and statistics).

##### Accessibility

Due to different response categories a separate PCA analysis was carried out for eight items hypothesised to measure ease of access to different places. These places were based on similar destinations reported by Timperio et al. [35] although the stem asked was '*How easy or difficult to get to the following places*....' rather than 'within *walking distance*'. Children could select from four response options (Very Easy to Very Difficult). Items were reverse coded so that a higher score represented easier access. Two factors emerged a). **Access (1) **explaining 28.14% of the variance and included the destinations: local shops, big shopping centre, playground or open space, bus stop, sports centre and library and b). **Access (2) **explaining 11.11% of the variance and containing two items representing ease of access to school and best friend's house.

##### Distance

Straight line distance from home to primary school in metres was calculated for each participant from grid references derived from home and school postcodes using the National Statistics Postcode Directory. Potential confounders included in analyses were:

##### Daylight

Minutes of daylight available on the first day of measurement were determined from standard tables. The variable used was minutes of daylight from 3 pm until sunset as an indicator of available daylight after school.

##### Level of Deprivation

The UK Index of Multiple Deprivation (IMD) 2007score based on full home post-code was used as an index of neighbourhood deprivation for each child.

##### Pubertal status

Pubertal status was measured using the scale developed by Petersen et al. [36] and five derived stages (equivalent to Tanner stages) were used in analyses.

##### Body mass index (BMI)

Height (m) was measured using a stadiometer and weight (kg) was measured using digital scales (SECA) with children wearing indoor clothing, and shoes removed. Body Mass Index (BMI) was calculated as weight (kg)/(height (m))^2 ^and BMI Standard Deviation Score (SDS) was derived from standard tables [37].

Gender, date of birth and full post-code for participating children were confirmed by the Local Education Authority.

### Data analyses

Means, standard deviations and checks for normality were calculated for all perceptions of the environment. Differences in activity context between boys and girls were assessed by Χ^2 ^and differences in perceptions of the environment were assessed by independent t-tests. Spearman correlations were used to examine the relationship between physical activity contexts. Logistic regression models were used to examine associations between exposures (independent mobility and perceptions of the environment) and outcomes, outdoor play, exercise/sport or active travel. The physical activity contexts were coded as follows: For both play and ex/sport the reference group was those children that reported taking part ***less than everyday ***(Play: boys = 402(62.9%), girls = 476(71.8%); Ex/sport: boys = 164(25.7%), girls = 265(40.1%)). They were compared to those children who reported taking part ***everyday***. For mode of travel home from school, the reference group was travel by car (boys = 189(29.6%), girls = 166(25.1%)) and was compared to those who reported walking or going by bike (boys = 445(69.6%), girls = 492(74.4%)). Taking the bus/train home from school was excluded from analyses due to the small number of children reporting this travel mode (n = 8). All models were adjusted for potential confounders: hours of daylight, IMD, pubertal stage and BMI SDS. Where confounders were significantly related to the physical activity contexts, interaction terms with other significant exposures were entered into the model and F tests used to test the significance of the interaction. Due to the well documented gender differences in physical activity and independent mobility all regression analyses were carried out separately by gender [38,39]. Robust standard errors were used to account for the clustering of participants within schools. Principal components analyses were carried out using SPSS (version 14.0) and the remainder of analyses were carried out using STATA (version 10). Significance was set at p < 0.05.

## Results

Of the 1899 Year 6 children from the 23 schools invited to take part in the study, 1340 provided parental consent (70.5%). Of these 33 were absent on the days of measurement. Seven participants did not complete the computerised questionnaire leaving 1300 for analysis. The majority of the sample was white (83.3%) and due to the heterogeneity in the non-white participants (6.6% Black, 4.8% Asian, 4.7% mixed) ethnicity was not included as an exposure in analyses.

### Gender differences

Boys had generally more positive perceptions of the environment compared to girls (Table 1) but the difference only reached significance (p < 0.05) for **Local-IM, Area-IM**, perceptions of **personal safety **and** traffic safety**. Boys scores were significantly lower than girls on the **Constraint **scale indicating that boys felt more 'constrained' by their local environment or 'stuck inside' compared to girls. Boys reported taking part significantly more often in play and ex/sport compared to girls but there was no gender difference in usual school travel mode (Table 2). The mean distance from home to school was 828.96 (sd = 1036) metres with no significant gender difference.

**Table 2:** Gender differences in frequency of participation in physical activity contexts

	Travel home from school				
	Bus/train (n = 8)	Car (n = 315)	Walk (n = 936)	Cycle (n = 40)	**Χ**^**2**^, **p**
Gender	N (%)	N (%)	N (%)	N (%)	
Boys	5 (0.8)	159 (24.9)	445 (69.6)	30 (4.7)	
Girls	3 (0.5)	156 (23.6)	492 (74.4)	10 (1.5)	** -1.538 **p = 0.124
	**Frequency of outdoor play**				
	**<1-2 week** **(n = 122)**	**1-2 week** **(n = 292)**	**Most days** **(n = 464)**	**Everyday** **(n = 422)**	**Χ**^**2**^, **p**
**Gender**	N (%)	N (%)	N (%)	N (%)	
Boys	50 (7.8)	122 (19.1)	230 (36.0)	237 (37.1)	
Girls	72 (10.9)	170 (25.7)	234 (35.4)	185 (28.0)	-4.223 p < 0.001

	**Frequency of structured exercise/sport**				
	**<1-2 week** **(n = 32)**	**1-2/week** **(n = 85)**	**Most days** **(n = 312)**	**Everyday** **(n = 871)**	**Χ**^**2**^, **p**
**Gender**	N (%)	N (%)	N (%)	N (%)	
Boys	11 (1.7%)	28 (4.4)	125 (19.6)	475 (74.3)	
Girls	21 (3.2%)	57 (8.6)	187 (28.3)	396 (59.9)	-5.660 p < 0.001

### Correlates of physical activity context

The three physical activity contexts were weakly significantly correlated to each other. Higher frequency of self-reported outdoor play was more strongly related to higher frequency for structured ex/sport (r = 0.358, p = 0.001) than active travel from school (r = 0.084, p = 0.003). Structured ex/sport was weakly significantly positively correlated to mode of travel from school (r = 0.082, p = 0.003).

Results of the logistic regression analyses show that for outdoor play (Table 3), boys with greater independent mobility (**Local-IM and Area-IM**) and **Social Norm **scores were approximately one and a half times more likely to report playing out every day compared to those boys who played out less frequently. **Area-IM **and **Social Norm **were also positively related to playing out more often for girls along with perceptions of **traffic safety**. In contrast girls were less likely to play out every day if they had a higher (more positive) score for perceptions of **Neighbourhood Nuisance**. The significant model (p < 0.001) explained 8.8% (R^2 ^= 0.088) and 8.7% (R^2 ^= 0.087) of the variance for boys and girls respectively.

**Table 3:** Factors associated with likelihood of playing everyday using logistic regression modelling

	Boys (n = 631)				Girls (n = 639)			
	OR	SE	95%CI	P	OR	SE	95%CI	P
	***Likelihood of playing everyday***							
Local-IM	**1.58**	**0.228**	**1.19-2.10**	**0.002**	1.32	0.204	0.97-1.78	0.076
Area-IM	**1.49**	**0.194**	**1.16-1.93**	**0.002**	**1.47**	**0.236**	**1.08-2.02**	**0.015**
Aesthetics	0.90	0.121	0.69-1.17	0.437	1.16	0.180	0.86-1.57	0.312
Nuisance	0.93	0.148	0.68-1.27	0.665	**0.56**	**0.103**	**0.39-0.80**	**0.002**
Personal safety	0.90	0.183	0.70-1.43	0.991	0.91	0.172	0.62-1.31	0.603
Traffic safety	1.07	0.177	0.78-1.49	0.652	**1.63**	**0.301**	**1.14-2.34**	**0.008**
Social Norm	**1.63**	**0.312**	**1.12-2.37**	**0.010**	**1.53**	**0.324**	**1.01-2.31**	**0.044**
Access (1)	1.30	0.314	0.81-2.08	0.282	0.96	0.242	0.59-1.58	0.887
Accesss (2)	0.88	0.136	0.65-1.19	0.417	1.09	0.195	0.77-1.55	0.626
Playspace	1.08	0.162	0.81-1.45	0.604	1.34	0.239	0.95-1.91	0.092
Constraint	1.09	0.143	0.84-1.41	0.512	1.01	0.128	0.79-1.29	0.952
Distance	0.99	0.001	0.99-1.00	0.845	0.99	0.001	0.99-1.00	0.312
Daylight	0.99	0.001	0.98-1.00	0.982	1.00	0.001	0.99-1.01	0.648
SDSBMI	0.98	0.078	0.85-1.15	0.847	1.01	0.782	0.87-1.18	0.891
IMDscore	1.00	0.001	0.99-1.01	0.800	0.99	0.001	0.99-1.00	0.781
Pubertal status	1.08	0.126	0.86-1.35	0.512	1.11	0.127	0.89-1.39	0.359

SDSBMI: standardised body mass index, IMD: Index of Multiple Deprivation

Reference group: Playing less than everyday

**Bold **text indicates significant relationship

With the exception of **Local-IM **for boys, different environmental perceptions were related to the likelihood of taking part in structured ex/sport sport everyday compared to those found for play (Table 4). For boys, greater scores for **Local-IM, Personal Safety **and easier access to facilities (**Access 1**) were related to exercising or doing sport every day. For girls, exercising or playing sport every day was related to easier access to both school and friend's house (**Access 2**), a broader range of facilities (**Access 1**) and more space to play both inside and outside the home (**Playspace**). As a greater standardised BMI was related to a significantly decreased likelihood of taking part in structured exercise/sport for boys, a test for interaction was carried out with other significant exposures. Interaction terms were however not significant (p > 0.05). The model (p < 0.001) explained 9.6% (R^2 ^= 0.096) and 6.5% (R^2 ^= 0.065) of the variance for boys and girls respectively.

**Table 4:** Factors associated with likelihood of taking part in structured exercise/sport everyday using logistic regression modelling

	Boys (n = 631)				Girls (n = 639)			
	OR	SE	95%CI	P	OR	SE	95%CI	P
	***Likelihood of taking part in structured exercise/sport everyday***							
Local-IM	**1.42**	**0.207**	**1.06-1.89**	**0.017**	1.22	0.159	0.95-1.58	0.122
Area-IM	1.34	0.211	0.98-1.82	0.065	1.08	0.156	0.82-1.44	0.570
Aesthetics	0.78	0.111	0.59-1.03	0.090	1.16	0.161	0.89-1.53	0.259
Nuisance	1.23	0.237	0.84-1.79	0.282	0.91	0.151	0.66-1.26	0.570
Personal safety	**1.47**	**0.318**	**1.02-2.24**	**0.049**	0.93	0.169	0.66-1.33	0.711
Traffic safety	0.79	0.151	0.55-1.15	0.226	0.82	0.138	0.59-1.15	0.254
Social Norm	1.30	0.240	0.90-1.86	0.161	1.37	0.263	0.95-2.00	0.095
Access (1)	**1.62**	**0.411**	**1.01-2.66**	**0.051**	**1.65**	**0.362**	**1.07-2.53**	**0.023**
Access (2)	1.27	0.218	0.91-1.77	0.165	**1.45**	**0.229**	**1.07-1.98**	**0.018**
Playspace	1.02	0.164	0.75-1.40	0.885	**1.41**	**0.219**	**1.04-1.91**	**0.025**
Constraint	0.79	0.104	0.61-1.02	0.077	0.80	0.097	0.63-1.01	0.066
Distance	1.00	0.001	0.99-1.00	0.417	0.99	0.001	0.99-1.00	0.917
Daylight	1.00	0.001	0.99-1.00	0.610	0.99	0.001	0.99-1.00	0.438
SDSBMI	**0.80**	**0.072**	**0.67-0.96**	**0.015**	0.99	0.068	0.87-1.14	0.972
IMDscore	1.00	0.006	0.99-1.01	0.815	099	0.005	0.98-1.00	0.141
Pubertal status	1.08	0.131	0.85-1.37	0.534	1.20	0.121	0.98-1.46	0.078

SDSBMI: standardised body mass index, IMD: Index of Multiple Deprivation

Reference group: taking part in structured exercise/sport < everyday

**Bold **text indicates significant relationship

For both boys and girls an increased likelihood of walking or cycling to school was associated with higher levels of **Local-IM **(Table 5). For boys, walkers and cyclists were also more likely to report positive perceptions of access to school and friends house (**Access 2**) and other destinations (**Access 1**). Objectively measured distance (m) from home to school was a significant predictor for both boys and girls, with those living further away less likely to actively commute home from school. For girls only, increased likelihood of walking or cycling to school was also associated with more negative perceptions of **Neighbourhood Nuisance**. As greater deprivation was related to a significantly decreased likelihood of walking or cycling to school for both boys and girls, a test for interaction was carried out with other significant exposures. Interaction terms were however not significant (p > 0.05). The model (p < 0.001) explained 22.4% (R^2 ^= 0.224) and 24.5% (R^2 ^= 0.245) of the variance for boys and girls respectively.

**Table 5:** Factors associated with likelihood of walking/cycling home from school using logistic regression modelling

	Boys (n = 631)				Girls (n = 639)			
	OR	SE	95%CI	P	OR	SE	95%CI	P
	***Likelihood of walking/cycling home from school***							
Local-IM*	**1.41**	**0.201**	**1.07-1.87**	**0.014**	**1.49**	**0.251**	**1.07-2.07**	**0.018**
Area-IM	1.03	0.167	0.75-1.42	0.833	0.91	0.169	0.64-1.31	0.617
Aesthetics	0.93	0.165	0.65-1.31	0.682	1.04	0.162	0.77-1.41	0.798
Nuisance	0.68	0.151	0.44-1.05	0.084	**0.61**	**0.127**	**0.40-0.91**	**0.017**
Personal safety	0.83	0.208	0.51-1.35	0.462	1.35	0.299	0.88-2.08	0.172
Traffic safety	1.01	0.233	0.64-1.59	0.995	0.72	0.127	0.51-1.01	0.064
Social Norm	0.89	0.190	0.59-1.36	0.462	1.07	0.273	0.65-1.77	0.790
Access(1)	**1.87**	**0.557**	**1.04-3.36**	**0.035**	1.00	0.281	0.58-1.74	0.989
Access (2)	**2.07**	**0.460**	**1.34-3.21**	**0.001**	1.43	0.322	0.92-2.23	0.110
Playspace	0.92	0.165	0.65-1.31	0.657	0.86	0.161	0.59-1.24	0.430
Constraint	1.04	0.180	0.74-1.46	0.804	1.22	0.178	0.92-1.62	0.173
Distance	**0.99**	**0.001**	**0.98-0.99**	**0.001**	**0.99**	**0.001**	**0.98-0.99**	**0.001**
Daylight	1.00	0.001	0.99-1.00	0.346	0.99	0.001	0.99-1.00	0.774
SDSBMI	1.04	0.109	0.84-1.27	0.725	0.84	0.077	0.70-1.01	0.059
IMDscore	**0.98**	**0.006**	**0.97-0.99**	**0.034**	**0.98**	**0.007**	**0.97-0.99**	**0.040**
Pubertal status	0.91	0.138	0.68-1.23	0.556	1.14	0.132	0.91-1.43	0.249

SDSBMI: standardised body mass index, IMD: Index of Multiple Deprivation

Reference group: those who reported being driven by car to school

**Bold **text indicates significant relationship

*Note for analyses for passive vs active travel Local-IM did not include item "allowed to walk/cycle to school" without an adult.

## Discussion

This study investigated whether independent mobility, perceptions of the environment and distance from home to school were related to children's self-reported physical activity in three different contexts - outdoor play, structured ex/sport and active commuting to school. Boys reported playing and taking part in structured ex/sport more often than girls, but there was no gender difference in active commuting. This highlights the importance of active commuting as a source of physical activity for girls and that increasing opportunities for outdoor play and structured sport and exercise in girls may be warranted.

The results show that overall environmental correlates related differentially according to activity context and by gender. A higher frequency of outdoor play was related to higher scores for Social Norm ('there were other children around to play with' or 'you often see children playing on the street') for both boys and girls. This is supported by qualitative studies in which parent's reported that having children to play with related to an increased likelihood of their children playing outside [17,40] and Jago et al. [17] reported that different friendship groups at school and home are key influences on the location and type of physical activity in which children engage.

Greater independent mobility (IM) was related to higher frequency of play for both boys and girls. This is consistent with other studies where greater freedom to play out unsupervised by an adult was linked to higher levels of children's outdoor play and that the dependence on a parent's availability to take children to play spaces was a significant barrier to their outdoor play [41,42]. Wen et al. [43] in one of the few other quantitative studies that has investigated IM in relation to outdoor play reported that those who were 'mostly allowed to walk on their own', near where they lived were more than two and a half times (OR 2.56, CI 1.84-3.58) more likely to spend at least 30 minutes outside after school compared to those who were never allowed to walk on their own near where they lived. Wen et al. [43] also reported that more positive perceptions of neighbourhood safety (measured by a single item) were related to higher levels of outdoor play. This study does not fully support this finding as personal safety was not a significant predictor of outdoor play. However more positive perceptions of traffic safety (pollution, traffic density, crossing places) were related to higher frequency of girls play. Comparative data relating children's perceptions to play is limited but studies investigating parents perceptions show that higher density and speed of traffic inhibits their willingness to let their children play outdoors, particularly unsupervised [40,41].

Higher levels of IM were also related to higher levels of active commuting but this was only significant for males. This is similar to other UK data where children who were rarely or never allowed to go outside without an adult to walk to school or for leisure were twice as likely to be driven to school [44]. Similarly in Australia, children with lower levels of independent mobility to walk alone in their neighbourhood were less likely to walk to school [45]. Thus, there is growing evidence that higher independent mobility is an important correlate of active commuting in children of this age [20]. This may be because parents' direct involvement in active commuting with their child is less common at this age.

Consistent with other studies [20,46] a longer route from home to school was significantly related to decreased likelihood of active commuting to school. For boys, perceived ease of accessibility to school and wider destinations remained significant in the model. This supports other studies which show that both objective and perceived measures of the environment relate to active commuting in children [46,47]. Ease of access to a range of facilities was also a significant correlate of structured ex/sport, particularly for girls, which is consistent with other studies where access to facilities is related to participation in structured physical activity [38]. Independent mobility in the local (Local-IM) and wider area (Area-IM) was also a significant correlate for structured ex/sport but only for boys. This may be because boys range further than girls so are able to access facilities for structured sport and exercise unsupervised [42].

The strengths of this study include the measurement of three distinct physical activity contexts and environmental correlates in the same study allowing the relative importance of the different correlates to be investigated. The weak but significant positive correlations between the three physical activity contexts supports the view that children's participation is often specific to a particular physical activity context and that this specificity needs to be considered in the design of interventions as changes to increase physical activity in one context may not necessarily transfer to another [5]. The finding that different correlates generally relate to different physical activity contexts has been found in a small number of other studies [22,48] but this study includes a wider, more robustly measured range of correlates and adjusts for powerful confounders in analyses. Although this study has measured specific activity contexts in line with the recommendations by, for example Giles-Corti et al. [5], future work should measure both context specific behaviours alongside context specific correlates. Some progress in this area has been made with active commuting where studies have included some measures specifically tailored to active commuting in children [34,42,46] but there are few measures available to investigate the specific correlates of children's outdoor play.

The finding here that independent mobility was the only correlate related to all three physical activity contexts adds to the recent literature on independent mobility and physical activity in children [25,40,42]. Further work investigating independent mobility is warranted as this has been in decline over recent decades [24]. This cross-sectional study cannot determine the direction of relationship or causality. Longitudinal data is required to indicate whether high independent mobility is a precursor to or a consequence of higher physical activity levels, whether change in independent mobility is related to change in specific physical activity behaviours and whether independent mobility is temporally linked to other perceived and objective measures of the environment. Prezza and Pacilli [38] showed that greater independent mobility and associated play in public areas during childhood was related to a stronger sense of community, and less fear of crime. Temporal data are required to determine if independent mobility and children's consequent familiarity with their environment leads to more positive perceptions of their environment or whether positive perceptions are a precursor of children's desire and parent's willingness to afford greater independent mobility [41]. Further work should also consider factors not included here, such as car ownership, as it may moderate the relationships between independent mobility, perceptions of the environment and participation in different physical activity contexts, particularly in more rural settings. Also due to the relatively small number of participants from minority ethnic groups in this sample, ethnicity was not included in the analysis. Further work in a more diverse sample is required to determine if participation in different physical activity contexts varies by ethnic group.

Longitudinal data are also important to determine how the relationship between independent mobility, environmental perceptions and participation in different activity contexts change over time. For example, Sener et al. [22] reported that participation in unstructured free play decreased with age whereas participation in structured out of home activities was higher in older (12 to 15 years) compared to younger children (5-11 years). Further work should also investigate both child and parental perceptions of the environment in relation to children's participation in different physical activity contexts as these may exert independent and interactive effects. Whilst parents are still important gatekeepers of children's physical activity opportunities children's perceptions may increasingly influence their physical activity behaviour as they age.

## Conclusion

Different environmental correlates are related to boys and girls participation in different physical activity contexts. Interventions designed to increase physical activity levels should specify the activity contexts in which they seek to intervene, as well as identify and manipulate specific correlates that relate to these activity contexts. Children with higher levels of independent mobility report higher participation across a range of physical activity contexts.

## Competing interests

The authors declare that they have no competing interests.

## Authors' contributions

Angie Page drafted the initial manuscripts and conducted the analyses. All other authors contributed to the design of the project and the writing of the manuscript. All authors read and approved the final manuscript.
